# The Combination of Sulforaphane and Fernblock^®^ XP Improves Individual Beneficial Effects in Normal and Neoplastic Human Skin Cell Lines

**DOI:** 10.3390/nu12061608

**Published:** 2020-05-30

**Authors:** Simona Serini, Roberta Guarino, Renata Ottes Vasconcelos, Leonardo Celleno, Gabriella Calviello

**Affiliations:** 1 Department of Translational Medicine and Surgery, Division of General Pathology, School of Medicine, Università Cattolica del S. Cuore, L.go F. Vito, 1 00168 Rome, Italy; simona.serini@unicatt.it (S.S.); robertagua92@hotmail.it (R.G.); 2 Fondazione Policlinico Universitario A. Gemelli IRCCS, Largo A. Gemelli, 8 00168 Rome, Italy; lcelleno@gmail.com; 3 Centro de Investigação Translacional em Oncologia, Laboratório de Oncologia Experimental, Departamento de Radiologia e Oncologia, Instituto do Câncer do Estado de São Paulo, Faculdade de Medicina da Universidade de São Paulo, Dr. Arnaldo Avenue, 251-Cerqueira César, São Paulo 01246000, Brazil; renataottes@yahoo.com.br; 4 Clinical Dermatology, School of Medicine, Università Cattolica del S. Cuore, L.go F. Vito, 1 00168 Rome, Italy

**Keywords:** melanoma cells, keratinocytes, Fernblock^®^ XP, sulforaphane, migration, invasion, metalloproteinases, antioxidant

## Abstract

Plenty of evidence supports the health effects exerted by dietary supplements containing phytochemicals, but the actual efficacy and safety of their combinations have been seldom experimentally evaluated. On this basis, we investigated in vitro the antioxidant/antineoplastic efficacy and anti-aging activity of a dietary supplement containing sulforaphane (SFN), a sulfur-isothiocyanate present in broccoli, combined with the patented extract Fernblock^®^ XP (FB), obtained from the tropical fern *Polypodium leucotomos*. We evaluated the effect of SFN and FB, alone or in combination, on migration ability, matrix metalloproteinases (MMP) production, neoangiogenic potential and inflammasome activation in human WM115 and WM266-4 melanoma cells. Moreover, the effects on MMPs and reactive oxygen species production, and IL-1β secretion were studied in human normal keratinocytes. The SFN/FB combination inhibited melanoma cell migration in vitro, MMP-1, -2, -3, and -9 production, inflammasome activation and IL-1β secretion more efficiently than each individual compound did. In normal keratinocytes, SFN/FB was more efficient than SFN or FB alone in inhibiting MMP-1 and -3 production and IL-1β secretion in the presence of a pro-inflammatory stimulus such as TNF-α. The potential use of SFN/FB based supplements for the prevention of skin aging and as adjuvants in the treatment of advanced melanoma is suggested.

## 1. Introduction

Complex extracts or single compounds derived from plants have attracted considerable attention as potential preventive or therapeutic agents in chronic diseases pathogenically related to oxidative stress and/or inflammation. Currently, the main challenge is to identify intracellular molecular target(s) that may be regulated by these naturally derived products and that may specifically explain their health effects. Interestingly, it has been observed that many of the molecular targets identified play key roles in normal (such as cell differentiation, death and survival) or pathological processes (such as cellular stress and inflammatory responses) [1,2,3,4]. On these bases, it has been suggested that these natural products could be used as powerful therapeutic agents for innovative and targeted therapy in chronic inflammatory or neoplastic diseases [5].

It should be underlined that the compounds showing antioxidant properties (either endogenous or diet-derived) once inside the cells do not work independently, but as components of a complex organization, where each compound plays a specific role, and no one can perform the function of the entire team. For this reason, supplementing a single antioxidant may alter the natural balance of antioxidants in tissues, and even produce paradoxical pro-oxidant effects [6]. Thus, a balanced diet rich in vegetables has been usually proposed as a much better solution than the supplementation of a single bioactive compound. However, an alternative strategy has been also proposed [7], which consists in preparing supplements including combinations of phyto-derived extracts or compounds with different properties. The aim is to obtain multifunctional supplements able to integrate diets that, especially in some physiological (pregnancy, growth, exercise, aging) or pathological conditions (chronic inflammations, cancer, neurodegenerative disorders, etc.), do not satisfy the increased/changed body requirements. Several of these combined supplements have been already put on the market, with the assumption that, since each individual component is known to induce some beneficial effects, the efficacy of their combination cannot be other than increased. The current worldwide legislation generally endorses this quite empiric approach, by considering dietary supplements just as food. This means that preventive experimental investigations are usually not required to understand which is the actual effect (either synergistic, additive, or antagonistic) when the different ingredients are combined in a single dietary supplement. Consequently, a quite exiguous number of experimental works have been so far performed to investigate the actual antioxidant or anti-inflammatory effects of the combinations of ingredients that are present in a dietary supplement, either before or after their commercialization [8]. However, such kinds of study would be essential, for instance, to understand if the expected effects of a combination of ingredients are greater than that of each individual component alone. That is theoretically possible, since, for example, among the known antioxidants, some are hydrophilic and others are lipophilic, or differently compartmentalized inside the cells. Moreover, they could protect against specific types of reactive oxygen or nitrogen species or have either direct or indirect antioxidant action. Additionally, the different substances could exert beneficial effects by targeting specific and different cellular and molecular factors and pathways. Such studies on combined supplements could be essential to verify if the same effects usually obtained with high doses of one component could be equally obtained by combining lower doses of it with other components at relatively low doses, thus avoiding the potential cytotoxicity that can be associated to high doses. On the contrary, the combination could be found not to increase the beneficial effects of one of the components or even decrease its activity. This could indicate that its individual supplementation could be the better option and already enough to obtain the expected effect at the maximum level, and its combination with other substances useless or even detrimental. Overall, these findings could help producers to improve the health properties and reduce the potential adverse effects of a dietary supplement formulation.

Following our previous studies where we investigated [8,9] the antioxidant and anti-inflammatory effects of several combined dietary supplements in vitro, we have now evaluated the in vitro effects of one dietary supplement currently indicated for skin pathologies. This dietary supplement contains the patented extract (Fernblock^®^ XP) (FB) of the tropical fern *Polypodium leucotomos*, which is enriched with ferulic acid and chlorogenic acid, combined with sulforaphane (SFN), an isothiocyanate found at high levels in vegetables of the *Brassicaceae* family, such as broccoli or cauliflowers. Previously published reports [10,11,12,13,14] showed that, when individually administered, Fernblock^®^ XP or SFN possessed antioxidant and anti-inflammatory activities in skin disorders and other pathological conditions. Moreover, SFN had shown powerful antineoplastic effects in different cancer cell lines, including melanoma [15,16,17,18,19,20]. We administered the individual supplement components, or the combination of different concentrations of them, to normal and neoplastic human skin cell lines (human immortalized keratinocytes at different degree of differentiation, and human melanoma cells showing different degree of malignity), and investigated if and at what extent their antioxidant, anti-inflammatory and anti-tumor effects could be changed by their combination.

## 2. Materials and Methods

### 2.1. Cell Lines and Treatments

The human primary melanoma (WM115) and metastatic melanoma (WM266-4) cell lines were kindly provided by Dr. M.C. Failla (Istituto Dermopatico dell’Immacolata, Rome, Italy). Cells were grown in DMEM) medium containing glutamine, non-essential amino acids, sodium pyruvate and fetal bovine serum (FBS, 10%).

The human immortalized keratinocytes HaCaT and NCTC2544 were obtained by the American Type Culture Collection (ATCC, Rockville, MD, USA) and by Dr. R. De Bellis (Università di Urbino, Italy), respectively and were grown in DMEM medium supplemented with glutamine, antibiotics (100 U/mL penicillin and 100 μg/mL streptomycin) and 10% FBS. All cell lines were maintained at 37 °C in humidified atmosphere at 5% CO_2_.

Sulforaphane (DL-Sulforaphane, SFN) was obtained by Sigma-Aldrich (S4441, Sigma, St. Louis, MO, USA) and has been added to the culture medium from a DMSO stock solution. Control cells were treated with the same amount of vehicle alone and the final DMSO concentration in the culture medium never exceeded 0.5% (vol/vol).

The *Polypodium leucotomos* extract, Fernblock^®^ XP (FB), was kindly provided by Difa Cooper (Varese, Italy). Previous studies have determined the chemical composition of *Polypodium leucotomos* extracts and have identified p-coumaric, ferulic, caffeic, vanillic and chlorogenic acids as the major phenolic compounds [21]. FB was solubilized in the culture medium at the concentration of 5 mg/mL. Then, 200 μL/mL or 400 μL/mL of this stock solution was added to the culture medium in order to obtain the final concentrations of 1 and 2 mg/mL.

We used the above reported concentrations for FB and SFN since in preliminary experiments (not shown) performed by using the Trypan blue dye exclusion method (see below) we observed that the percentage of non-viable cells could be maintained equal or inferior to 5% in the presence of SFN (added to all the cells, alone or in combination with FB) only if it was used at concentrations ≤ 10 µM; and in the presence of FB (added to all the cells, alone and in combination with SFN), only if used at concentrations ≤ 2 mg/mL

### 2.2. Cell Growth Evaluation

WM115 and WM266-4 melanoma cells were seeded in 24-well multi-well culture plates at a concentration of 2 × 10^4^ cells/well. After 24 h, culture medium was removed and replaced with fresh culture medium containing, or not, SFN at two different concentrations (5 and 10 µM) and/or FB extract at two different concentrations (1 and 2 mg/mL), given alone or in combination. At the times indicated (24–72 h), culture medium was removed and cells were tripsynized and centrifuged at 1200× *g* rpm for 5 min. Cells were then counted using a hemocytometer Neubauer chamber. Cell viability was evaluated by the Trypan blue dye exclusion method.

### 2.3. Cell Migration Assay

Cell migration has been analyzed by using the Wound Healing assay in vitro. To this aim, disposable silicon inserts (Ibidi, Munchen, Germany) were used. The inserts consist of two chambers separated by a 500 μm width silicon sept. The inserts were put into 6-well multi-well culture plates and 70 µL of WM115 and WM266-4 melanoma cell suspensions (at a concentration of 5 × 10^5^ and 3 × 10^5^ cells/mL, respectively) were put into each chamber of the silicon inserts. Culture medium not containing cells (1.3 mL) was put in the wells outside the silicon inserts. After 24 h, the silicon inserts were removed, and cell monolayers separated by a 500 μm cell free gap were obtained. Samples were photographed (representing the time 0) and then cell culture medium was replaced by fresh culture medium containing, or not, SFN (5 and 10 μM) and FB (1 and 2 mg/mL) given alone and/or in combination. The ability of cells to migrate was analyzed at the indicated time points (24, 48 and 72 h) by evaluating the residual cell free area in the wells. The cultures were observed under a light microscope and photographed to save images, that were then analyzed by TScratch software (developed by the group of Dr. Koumoutsakos (CSE Lab), at the Eidgenössische Technische Hochschule (ETH), Zurich, Switzerland) [22].

### 2.4. ELISA Analysis of Matrix Metalloproteinases MMP, VEGF and IL-1β Production

WM266-4 and NCTC 2544 cells were seeded in a 96-well multi-well culture plate at a concentration of 5 × 10^4^ cells/well. After 24 h, cell culture medium was removed and replaced with fresh culture medium containing, or not, SFN (5 and 10 μM) and FB (1 and 2 mg/mL), given alone and/or in combination for WM266-4 cells and in the presence of TNF-α (20 ng/mL) for NCTC 2544 cells. After 16 h in the presence of TNF-α, SFN (10 μM) and FB (1 and 2 mg/mL), alone and/or in combination, were added to the culture medium of NCTC 2544 cells for a further 18 h. After treatment (24 h for WM266-4 cells), cell supernatants were collected, centrifuged to remove suspended cell debris and stored at −20 °C until analysis. The amount of the different metalloproteinases secreted by WM266-4 and NCTC 2544 cells in the culture medium, as well as the amount of VEGF and IL-1β, was analyzed by commercial ELISA kits (MMP-1 human ELISA kit, CEK1274, Cohesion Biosciences, London, UK; MMP-2 Human ELISA kit, KA0391, Abnova, Heidelberg, Germany; MMP-3 Human ELISA kit, CEK1280, Cohesion Biosciences, London, UK; Human MMP-9 ELISA kit, CSB-E08006h, Cusabio, Wuhan, China; VEGF human ELISA kit, ENZ-KIT156-000, Enzo Life Sciences, Farmingdale, NY, USA; IL-1β human ELISA kit, NB-15-0176, NeoBiotech Clinisciences, Guidonia Montecelio, Rome, Italy), following the manufacturer’s instructions. The minimum detectable amounts of MMP-1, MMP-2, MMP-3, MMP-9, VEGF and IL-1β were: 20 pg/mL; 10 pg/mL; 15 pg/mL; 0.280 ng/mL, 4.712 pg/mL and 2.35 pg/mL, respectively.

### 2.5. Western Blot Analysis of NLRP3, ASC, Cleaved Caspase-1 and IL-1β

WM266-4 cells were seeded in 100 mm Petri dishes (3 × 10^5^ cells/mL). After 24 h, culture medium was removed and replaced with fresh culture medium containing, or not, SFN (10 µM), FB (1 mg/mL), alone and in combination. After 48 h, cells were trypsinized and centrifuged at 1200× *g* rpm for 5 min. Whole cell extracts were prepared according to Serini et al. [23]. 50 µg of proteins were separated on a 8% (for NLRP3 analysis) or 15% (for ASC, cleaved caspase-1 and IL-1β analysis) sodium dodecyl sulfate polyacrylamide gel and electroblotted on a nitrocellulose membrane. The membrane was blocked overnight at 4 °C with 5% dried milk (wt/vol) in PBS plus 0.05% Tween 20 and then incubated with specific antibodies to NLRP3 (MAB7578, clone #768319, R&D Systems, Minneapolis, MN, USA), ASC (clone N-15-R: sc 22514-R, Santa Cruz Biotechnology, Santa Cruz, CA, USA), cleaved caspase-1 (clone C-20: sc-515, Santa Cruz Biotechnology), IL-1β (clone H-153: sc-7884, Santa Cruz Biotechnology). As loading controls, the blots were reprobed with anti-β-actin (clone AC40, catalog # A-4700, Sigma-Aldrich) or anti-α-actinin (clone B-12, catalog # sc-166524, Santa Cruz Biotechnology) antibodies at a 1:1000 dilution. Following incubation with secondary rabbit (for ASC, cleaved caspase-1 and IL-1β), rat (for NLRP3), or mouse (for α-actinin and β-actin) antibodies, the immunocomplexes were visualized using the enhanced chemiluminescence detection system (GE Healthcare Life Sciences, Pittsburgh, PA, USA) and quantitated by densitometric analysis.

### 2.6. Evaluation of ROS Production

HaCaT and NCTC 2544 keratinocytes were seeded in 6-well multi-well culture plates at a concentration of 5 × 10^5^ cells/well. After 24 h, culture medium was removed and replaced with fresh culture medium containing, or not, SFN and/or FB. In this series of experiments, SFN was used at the concentrations of 2.5, 5 and 10 µM, since preliminary experiments had shown that higher concentrations induced necrotic effects in the cells (data not shown).

After a 24 h treatment, culture medium was removed, cells were trypsinized and centrifuged. 5 × 10^5^ cells for each sample were re-suspended in phosphate buffered saline (PBS) containing the fluorogenic substrate 6-carboxy-2′,7′-dihydro-dichlorofluorescein diacetate (DCF, 50 µM). 150 µL of each sample were put in a 96-well multi-well culture plate and incubated at 37 °C in the dark for 30 min. The, fluorescence was measured by a plate cytofluorimeter [Cytofluor 2300/2350 Fluorescence Measurement System (Millipore Corp., Bedford, MA, USA)] with an excitation wavelength of 504 nm and an emission wavelength of 529 nm. Then, as a pro-oxidant stimulus, 100 µM H_2_O_2_ was added to each well, the plate was incubated at 37 °C in the dark for further 15 min and fluorescence was measured again.

### 2.7. Analysis of FB and SFN Additive or Synergistic Effects

The potential synergistic effects of the SFN and FB combined treatments was analyzed by their Combination Index (CI). CI was calculated using the Bliss Independence Model [24]. According to this method, the observed combination effect of two compounds (E_AB_) (% effect vs. control) was expressed as a probability (0 ≤ E_AB_ ≤ 1) and was compared to the expected additive effects of the single compounds expressed as: E_A_ + E_B_ (1 - E_A_) = E_A_ + E_B_ – E_A_E_B_, where 0 ≤ E_A_ ≤ 1 and 0 ≤ EB ≤ 1. The CI was calculated by using the following formula: CI= [(E_A_ + E_B_) − E_A_E_B_]/E_AB_. The effect of the combination of compounds was considered synergistic when CI < 1, additive when C = 1 and antagonistic when C > 1.

### 2.8. Statistical Analysis

Results are expressed as means ±SD and were analyzed by the One-Way Analysis of Variance (ANOVA), followed by Tukey’s test (InStat GraphPad Software).

The significance of interaction between FB and SFN (obtained by calculating the CI according to the Bliss Independence Model described in Paragraph 2.7) was evaluated by 2 × 2 factorial ANOVA of the individual and combination effects (Prism GraphPad software).

## 3. Results

### 3.1. Effect of SFN/FB Combination on Melanoma Cell Growth

**Figure 1:**
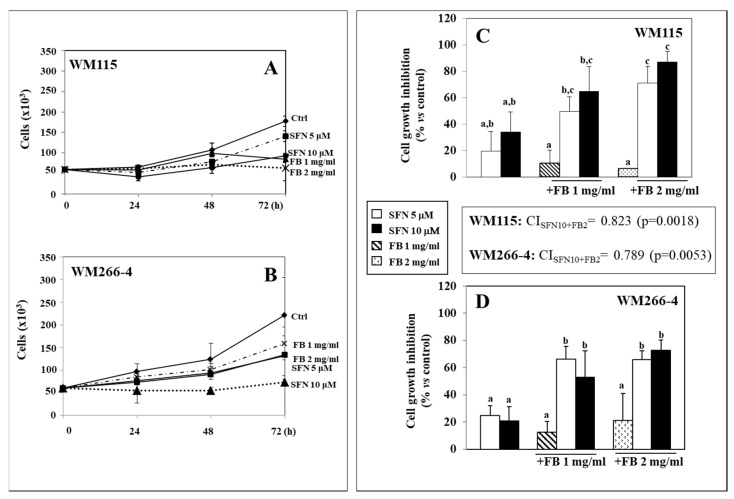
Effect of increasing concentrations of sulforaphane (SFN) (5 and 10 µM) and Fernblock^®^ XP (FB) (1 and 2 mg/mL) for different periods of time (24–72 h) in WM115 (**A**) and WM266-4 (**B**) human melanoma cells. In panels (**C**,**D**), the effect of a 24 h combined treatment with SFN and FB in WM115 (**C**) and WM266-4 (**D**) cells. In panels (**C**,**D**), values not sharing the same superscript letter are significantly different (*p* < 0.05, One-way ANOVA followed by Tukey’s test). In the box between panels (**C**,**D**) is shown the combination index (CI) obtained by treating WM115 and WM266-4 melanoma cells with the highest concentrations of SFN (10 μM) and FB (2 mg/mL) alone and in combination for 24 h. The interaction was considered significant when *p* < 0.05 (2 × 2 factorial ANOVA) (see Materials and Methods section for details).

We investigated whether the effects exerted by individual administrations of SFN or FB to melanoma cells and normal keratinocytes in vitro could be enhanced if the cells were simultaneously exposed to both the compounds. Figure 1A,B show the effect of SFN (5 and 10 µM) and FB (1 or 2 mg/mL) administered individually for increasing periods of time (0–72 h) to WM115 (Panel A) and WM266-4 (Panel B) melanoma cells. Both the compounds were able to inhibit the growth of melanoma cells in a dose- and time-dependent manner. We chose these concentrations since preliminary experiments performed by Trypan blue dye exclusion method had shown that they did not reduce cell viability more than 5% (data not shown). Figure 1C,D show the effect of a 24 h-treatment with combinations of SFN (5 and 10 µM) and FB (1 or 2 mg/mL) on WM115 (Panel C) and WM266-4 (Panel D) cell growth. We observed that the WM266-4 metastatic cells grew faster than the counterpart (WM115 cells) originated by the primary tumor, and that the separate addition of either SFN or FB reduced to a similar extent the growth of both the melanoma cell lines, independently from the degree of malignity. Interestingly, combinations of SFN and FB (SFN/FB) were able to inhibit significantly (*p* < 0.05) the growth of both the human melanoma cell lines more than each of the individual substances (Figure 1D). However, in WM115 melanoma cells, it was necessary to add the highest concentration of FB (2 mg/mL) to SFN (either 5 or 10 μM) to obtain an effect significantly higher (*p* < 0.05) than that observed with SF alone (Figure 1C). This finding suggests that the more aggressive metastatic WM266-4 cells may be more sensitive to the growth-inhibiting effect of the SFN/FB combination. In the box between panels C and D is reported the CI value obtained when both the cell lines were treated with the highest concentrations of SFN (10 μM) and FB (2 mg/mL), alone and in combination. As is shown in the box in both cell lines the CI value is <1 (CI_SFN10 + FB2_ = 0.823 and CI_SFN10 + FB2_ = 0.789 in WM115 and WM266-4 cells, respectively), indicating that the effect of the combination SFN/FB on melanoma cell growth can be considered synergistic. It is worth underlining that, also when using the SFN/FB combinations at the highest concentration analyzed here, we did not find any significant increase in the percentage of necrotic cells (data not shown), that remained always below the value of 5%, suggesting that the effect of the compounds on melanoma cell growth are not related to a cytotoxic and nonspecific effect, but presumably to the inhibition of cell proliferation or the induction of apoptosis.

### 3.2. Effect of SFN/FB Combination on Melanoma Cell Migration In Vitro

**Figure 2:**
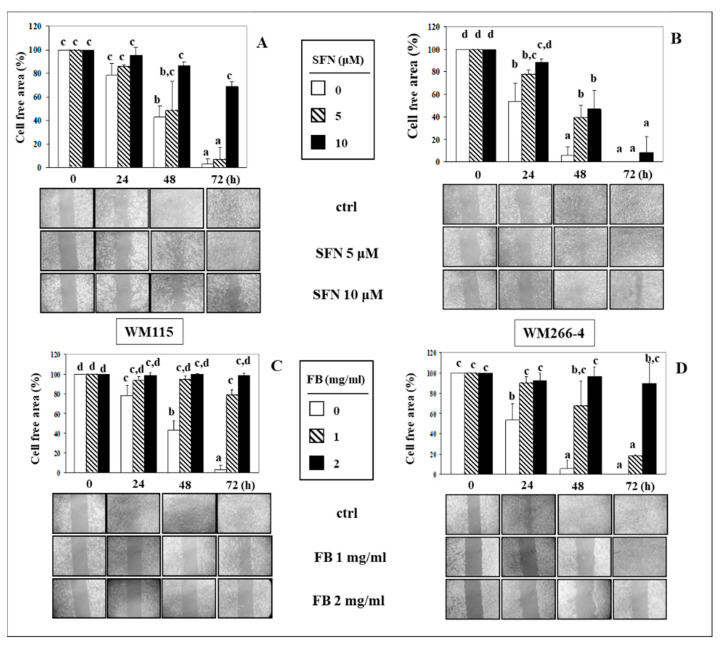
Effect of SFN and FB on migration ability evaluated in WM115 (**A**,**C**) and WM266-4 (**B**,**D**) melanoma cells by Wound Healing assay (see Materials and Methods section for details). Panels (**A**,**B**): cells were treated with SFN 5 and 10 µM for increasing periods of time (24–72 h); panels (**C**,**D**): cells were treated with FB 1 and 2 mg/mL for increasing periods of time (24–72 h). Values not sharing the same superscript letter are significantly different (*p* < 0.05, One-way ANOVA, followed by Tukey’s test). Below each histogram a representative Wound Healing assay of at least three different experiments is shown.

We also evaluated how the migration in vitro of WM115 and WM266-4 melanoma cells could change following their exposure to SFN and FB alone or in combination. Cells were treated with SFN (5 and 10 µM) (Figure 2A,B) and FB (1 and 2 mg/mL) (Figure 2C,D) for increasing periods of time (0–72 h). Their migration ability was evaluated as the decrease in the residual cell free area (wound area) present in the wells by using the Wound Healing assay in vitro. In control conditions, a gradual decrease of the wound area was observed for both the WM115 (Figure 2A) and WM266-4 (Figure 2B) cells over the 72 h incubation period, even though the decrease was faster in WM266-4, in agreement with their metastatic origin. At 72 h, the wound area was not present any more in both the melanoma cell cultures. The administration of either 10 µM SFN (but not 5 µM) or 1 and 2 mg/mL FB to WM115 cells for 48 or 72 h markedly and significantly (*p* < 0.05) inhibited cell migration (Figure 2A,C) and the cell ability to fill the wound area. On the other hand, at 48 h, SFN inhibited only partially the high migration ability of the highly invasive WM266-4 cells that completely filled the wound area after 72 h (Figure 2B). The WM266-4 cells also showed a lower sensitivity than the WM115 cells to the anti-migration effect of FB (Figure 2D). In this case, only the addition of the highest concentration of FB (2 mg/mL) was able to significantly (*p* < 0.05) suppress WM266-4 migration until 72 h.

**Figure 3:**
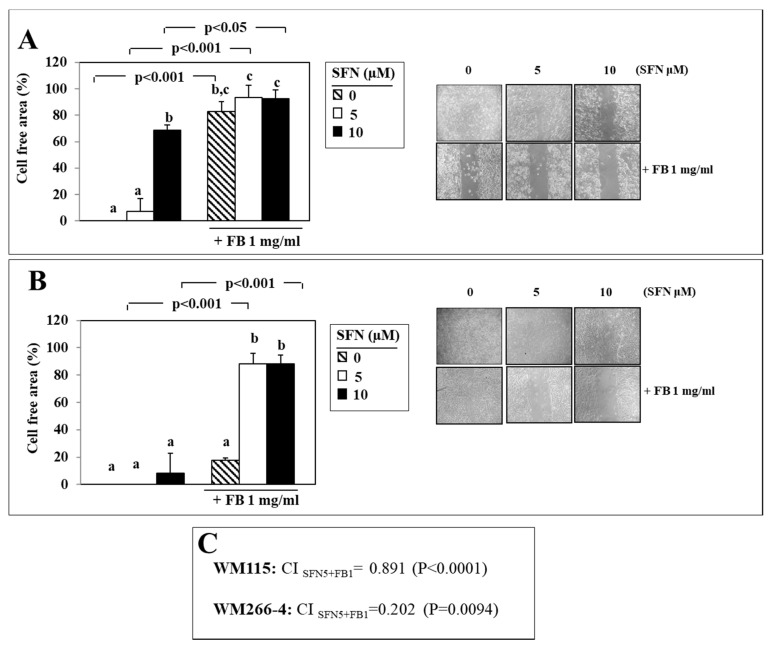
Effect of the combination of SFN (5 and 10 µM) and FB (1 mg/mL) on migration ability evaluated in WM115 (**A**) and WM266-4 (**B**) melanoma cells through Wound Healing assay (see Materials and Methods section for details) after 72 h of treatment. Values not sharing the same superscript letter are significantly different (*p* < 0.05, One-way ANOVA followed by Tukey’s test). Below each histogram a representative wound healing assay of at least three different experiments is shown. In panel (**C**) is shown the CI obtained by treating WM115 and WM266-4 melanoma cells with 5 μM SFN and 1 mg/mL FB, alone and in combination, for 72 h. The interaction was considered significant when *p* < 0.05 (2 × 2 factorial ANOVA) (see Materials and Methods section for details).

We chose the 72 h time point (when control cells completely filled the wound area) to evaluate the inhibitory effect of combinations of the two compounds on cell migration. We combined 1 mg/mL FB (since it was not able to inhibit cell migration at the maximal extent, like 2 mg/mL FB, see Figure 2) with either 5 µM or 10 µM SFN (Figure 3A,B). When FB was administered to WM115 cells in combination with either concentrations of SFN (Figure 3A), a marked inhibition of cell migration was observed (by 93.4% and 92.3%, respectively), that, however, was not significantly different from that observed when the cells were exposed to FB alone. On the contrary, neither SFN (5 µM or 10 µM) or 1 mg/mL FB administered alone were able to inhibit WM266-4 cell migration (Figure 3B), confirming that the invasive potential of these cells originally derived from metastasis was higher than that of the counterpart (WM115 cells) originated from the corresponding primary neoplasia in the same patient. Of interest, however, the combinations of the two substances, whichever SFN concentration was used, were always able to markedly inhibit the migration of these cells (1 mg/mL FB + 5 µM SFN: 88.1% inhibition; 1 mg/mL FB + 10 µM SFN: 88.2%) (Figure 3B). In box C is reported the CI value obtained when both the cell lines were treated with SFN (5 μM) and FB (1 mg/mL), alone and in combination. As is shown in the box, in both cell lines the CI value is <1 (CI_SFN10 + FB2_ = 0.891 and CI_SFN10 + FB2_ = 0.202 in WM115 and WM266-4 cells, respectively), indicating that the effect of the combination SFN/FB on melanoma cell migration observed at these concentrations can be considered synergistic.

### 3.3. Effect of SFN/FB Combination on Melanoma Cell MMP Production

**Figure 4:**
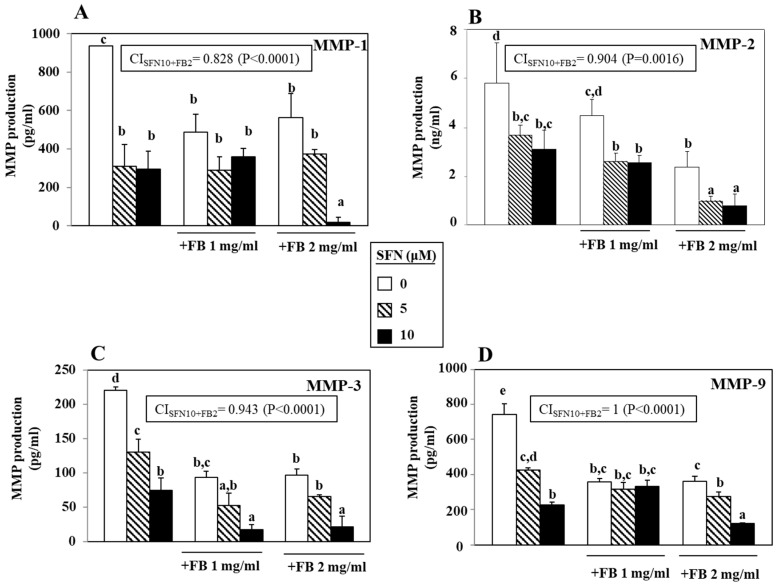
Effect of a 24 h-treatment with different concentrations of SFN (5 and 10 µM) and FB (1 and 2 mg/mL), alone or in combination, on the secretion of metalloproteinases (MMP)-1 (**A**), MMP-2 (**B**), MMP-3 (**C**) and MMP-9 (**D**) in WM266-4 melanoma cells. In each histogram, values not sharing the same superscript letter are significantly different (*p* < 0.05, One-way ANOVA, followed by Tukey’s test). Within each panel is shown the CI obtained following the treatment with SFN and FB at the highest concentrations (10 μM SFN and 2 mg/mL FB). The interaction was considered significant when *p* < 0.05 (2 × 2 factorial ANOVA) (see Materials and methods section for details).

An increased expression of several MMPs had been reported in highly invasive melanoma cells [25,26]. Since we showed a greater inhibitory effect of the FB/SFN combination on cell migration in the metastatic WM266-4 cell line, we evaluated if SFN and FB could alter the production of collagenase MMP-1, gelatinases MMP-2 and MMP-9 and stromelysin MMP-3 in this melanoma cell line (Figure 4). SFN and FB individually administered were both able to significantly inhibit the high WM266-4 cell production of MMP-1 (Figure 4A). SFN showed a tendency to exert a more powerful, even though not significant, inhibitory effect than that exerted by FB (5 and 10 µM SFN: 66.9% and 69% inhibition, respectively; 1 mg/mL and 2 mg/mL FB: 48% and 39.8% inhibition, respectively). Notably, when FB and SFN were added in combination at the highest concentrations (i.e., 2 mg/mL FB + 10 µM SFN), MMP-1 inhibition was markedly and significantly higher than that obtained when each single compound was administered alone (inhibition: 2 mg/mL FB + 10 µM SFN: 98%; 10 µM SFN and 2 mg/mL FB vs. 2 mg/mL FB + 10 µM SFN: *p* < 0.05 and *p* < 0.01, respectively).

The levels of MMP-2 secreted by WM266-4 cells were reduced by FB alone in a dose-dependent manner (1 and 2 mg/mL FB: 22.9% and 59.0% inhibition, respectively) (Figure 4B). In this case, the inhibitory effects of SFN (both at 5 and 10 µM) were higher than that observed with 1 mg/mL FB (inhibition 5 µM SFN: 36.8%; 10 µM SFN: 46.9%), and lower than that obtained with 2 mg/mL FB, even though in this case also the differences did not reach statistical significance. Instead, the combined treatment with FB (at both 1 and 2 mg/mL) plus SFN (at both 5 and 10 µM) was significantly (*p* < 0.05) more efficient than FB alone in inhibiting the MMP-2 production. However, the combination was significantly (*p* < 0.05) more efficient than SFN alone only when the higher concentration of FB was used (2 mg/mL). Again, the inhibitory effect exerted by the combination treatment on the MMP-3 production was more evident than that exerted by each individual agent (Figure 4C). However, the combination was significantly more efficient than that exerted by each of the two compounds alone only when the highest concentration of SFN (10 µM) was used in combination with either 1 or 2 mg/mL FB (SFN 10 µM vs. FB 1 mg/mL + SFN 10 µM, *p* < 0.05; SFN 10 µM vs. FB 2 mg/mL + SFN 10 µM, *p* < 0.05; FB 1 mg/mL vs. FB 1 mg + SFN 10 µM, *p* < 0.01; FB 2 mg/mL vs. FB 2 mg/mL + SFN 10 µM, *p* < 0.01). A similar trend of inhibition was observed also for MMP-9 secretion (Figure 4D). SFN was able to reduce in a dose-dependent manner the secretion of this MMP-9 (5 and 10 µM SFN: 42.5% and 69.6% inhibition, respectively, vs. control), while FB induced a similar inhibition at both the concentrations used (1 and 2 mg/mL FB: 52% and 51.4% inhibition, respectively, vs. control). However, in this case, the combination of the two highest concentrations of both FB and SFN was needed to induce a significantly higher reduction than those observed with each of them separately (10 µM SFN vs. 2 mg/mL FB + 10 µM SFN, *p* < 0.01; 2 mg/mL FB vs. 2 mg/mL FB + 10 µM SFN, *p* < 0.001). Overall, it can be concluded that a marked and significant inhibitory effect was observed for all the MMPs when SFN and FB were combined at the highest concentrations (10 μM and 2 mg/mL, respectively). Since in these experimental conditions the SFN/FB combination also reduced the cell number by 72.7% vs. control (see Figure 1D), it could be thought that its effect on the secretion of all the MMPs could be simply related to its growth inhibitory effect. However, it should be noted that the secretion of all the MMPs analyzed (and especially that of MMP-1,-2 and -3) was inhibited by the combination at a much higher extent (MMP-1: 98% inhibition vs. control; MMP-2: 86.5% vs. control; MMP-3: 90.3% inhibition vs. control; MMP-9: 83.8% inhibition vs. control). This demonstrates that at least a relevant part of the inhibitory effect of SFN/FB on the MMPs is dependent on a direct effect on the production of these enzymes and not just to the reduced number of cells. In each panel of Figure 4 is shown the respective CI obtained when WM266-4 cells were treated with the highest concentrations of SFN (10 μM) and FB (2 mg/mL). As it can be observed, the CI value in this condition is <1 for MMP-1, MMP-2 and MMP-3 (0.828, 0.904, 0.943, respectively). These results indicate a synergistic inhibitory effect of SFN and FB, suggesting a potential anti-invasive use for the SFN/FB combination in melanoma cells. On the contrary, in the same conditions, we observed for MMP-9 a CI value of 1.02, suggesting only an addictive effect.

### 3.4. Effect of SFN/FB Combination on NLRP3 Inflammasome Activation and VEGF Production in Melanoma Cells

**Figure 5:**
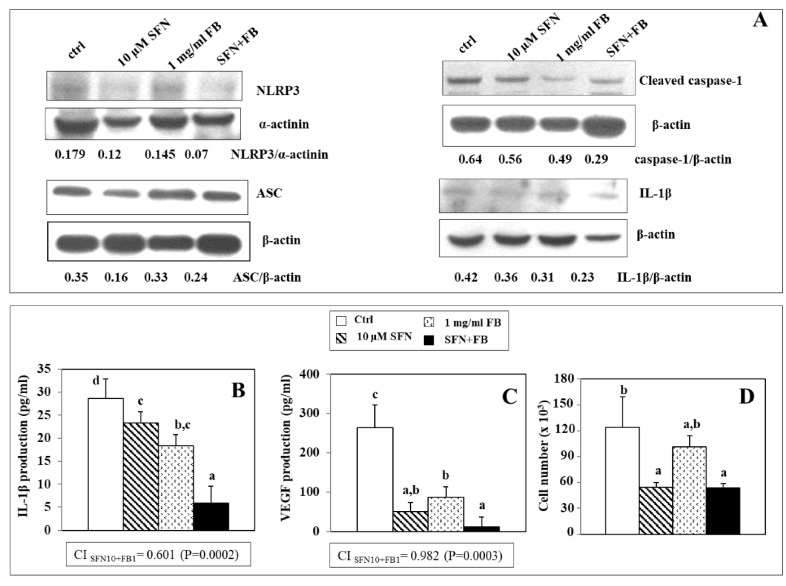
Effect of a combination of SFN (10 µM) and FB (1 mg/mL) on the expression of NLRP3, ASC, Cleaved caspase-1 and IL-1β (**A**) and on the secretion in the culture medium of IL-1β (**B**) and VEGF (**C**) and cell growth (**D**) in WM266-4 melanoma cells treated for 48 h. In panel A one representative Western blot of two similar experiments for each protein is shown. In panels (**B**–**D**) in each histogram, values not sharing the same superscript letter are significantly different (*p* < 0.05, One-way ANOVA, followed by Tukey’s test). In the boxes below panels (**B**,**C**), is shown the CI values obtained following the treatment of WM266-4 cells with SFN (10 μM) and FB (1 mg/mL), alone and in combination. The interaction was considered significant when *p* < 0.05 (2 × 2 factorial ANOVA) (see Materials and Methods section for details).

It was previously reported [27,28] that late stage human melanoma cells exhibit features of autoinflammatory diseases, since they constitutively express NLRP3 inflammasome components (NLRP3 and ASC) that are needed for the cleavage and activation of caspase-1, essential for the production of the pro-inflammatory cytokine IL-1β. For this reason, we evaluated the effect exerted by SFN and FB, administered individually or in combination (Figure 5), on the NLRP3 inflammasome components, as well as on the products of their activation (cleaved caspase-1 and IL-1β) in WM266-4 melanoma cells. We observed that SFN (10 µM) or FB (1 mg/mL) reduced the protein expression of NLRP3 and ASC, and correspondingly those of cleaved caspase-1, and IL-1β. Moreover, the simultaneous treatment with both SFN and FB induced an even more pronounced reduction of NLRP3, ASC, cleaved caspase and IL-1β (Figure 5A). SFN and FB, alone and in combination, significantly (*p* < 0.05) inhibited the expression IL-1β, as well as its secretion by WM266-4 cells in the culture medium (inhibition: SFN 10 µM 19%; 1 mg/mL FB 36%; SFN + FB 80%) (Figure 5B). However, the inhibition induced by the combination was significantly (*p* < 0.05) higher than that induced by each single compound (Figure 5B). Moreover, we evaluated also the effect of SFN/FB on VEGF secretion, since it was proposed [27] that in metastatic melanoma cells the activation of the inflammasome and the consequent IL1-β production may ultimately lead also to the increased production of VEGF. Melanoma cells exposed to SFN and FB exhibited a significantly decreased secretion of VEGF (*p* < 0.01) with respect to control cells (inhibition: SFN 10 µM 81%, FB 1 mg/mL 67.6%, SFN + FB 95%). In this case, however, the combination SFN/FB was significantly (*p* < 0.05) more efficient only with respect to FB alone (Figure 5C). It should be noted that at this time point (48 h, Figure 5D), 10 μM SFN (either alone or in combination with 1 mg/mL FB) inhibited cell growth by about 56% as compared to control, while FB alone did not significantly alter the cell number. However, the much more conspicuous inhibition that the SFN-FB combination induces on the IL-1β and VEGF secretion (80% and 95%, respectively, as compared to control) suggests that a considerable part of this can be related to the direct effect exerted by the compounds and not to the reduction of cell number. The boxes inside panels B and C show the CI obtained for the inhibition of IL-1β and VEGF expression following the treatment with SFN (10 μM) and FB (1 mg/mL), alone and in combination. The calculated CI values are both <1 (0.601 and 0.982 for IL-1β and VEGF, respectively), indicating a synergistic effect for SFN and FB, and suggesting a possible use of the SFN/FB combination as an inhibitor of the inflammatory microenvironment and neo-angiogenesis during the development and progression of melanoma.

### 3.5. Effect of SFN/FB Combination on Normal Keratinocyte MMP and IL-1β Production

**Figure 6:**
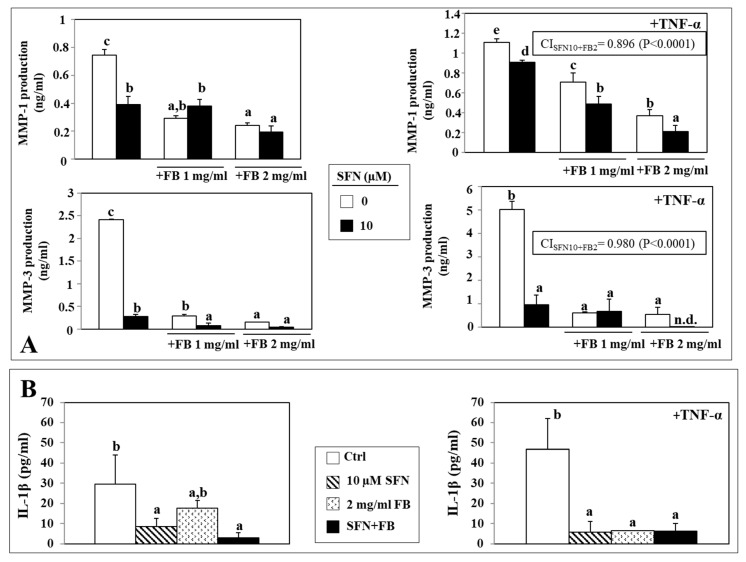
Effect of a 24 h-treatment with SFN (10 µM) and FB (1 and 2 mg/mL), alone and in combination, on the secretion in the culture medium of metalloproteinases (MMP)-1 and -3 (**A**) and of IL-1β (**B**) in NCTC2544 human keratinocytes in basal conditions and in the presence of TNF-α (20 ng/mL) as a pro-inflammatory stimulus. In each histogram, values not sharing the same superscript letter are significantly different (*p* < 0.05, One-way ANOVA followed by Tukey’s test). Within panel A (MMP-1 and MMP-3 in the presence of TNF- α is shown the CI obtained following the treatment with SFN and FB at the highest concentrations (10 μM SFN and 2 mg/mL FB). The interaction was considered significant when *p* < 0.05 (2 × 2 factorial ANOVA) (see Materials and Methods section for details).

Since during the development of skin aging an increased secretion of MMP-1 and MMP-3 has been observed, we evaluated if, also in normal keratinocytes, the combined SFN/FB treatment could inhibit the production of these MMPs more efficiently than the single compounds. We analyzed the production of MMPs in the keratinocyte cell line NCTC 2544, both at basal level and in the presence of the cytokine TNF-α (Figure 6), which was previously reported to activate the production of these MMPs in keratinocytes and cause skin damage [29].

In basal conditions, both 10 µM SFN and 1 or 2 mg/mL FB were able to significantly reduce MMP-1 production by NCTC 2544 keratinocytes (10 µM SFN: 47.5% inhibition; 1 and 2 mg/mL FB: 60.8 and 67.7% inhibition, respectively). The SFN/FB combined treatment, however, was not able to inhibit the MMP production more efficiently than FB alone (Figure 6A). Similarly, when keratinocytes were stimulated with TNF-α, SFN and FB alone exerted a significant inhibitory effect on MMP-1 production (10 µM SFN: 18% inhibition; 1 and 2 mg/mL FB: 36.4 and 66.7%, respectively, vs. control). However, in this case, the inhibitory efficacy of the combination on MMP-1 production was significantly higher than that of each compound given individually (10 µM SFN vs. 1 mg/mL FB + SFN 10 µM, *p* < 0.01; 10 µM SFN vs. 2 mg/mL FB + 10 µM SFN, *p* < 0.001; 1 mg/mL FB vs. 10 µM SFN + 1 mg/mL FB, *p* < 0.05; 2 mg/mL FB vs. 10 µM SFN + 2 mg/mL FB, *p* < 0.05) (Figure 6A). Therefore, we calculated the CI only for MMP-1 production in the presence of TNF-α, since only in this case was the SFN/FB combined addition able to significantly enhance the effect obtained by each of the individual compounds (i.e., with 10 μM SFN and 2 mg/mL FB), and we found a CI of 0.896, indicative of a synergistic effect.

Furthermore, we observed that the individual addition of 10 µM SFN markedly and significantly inhibited the production of MMP-3 in the same keratinocyte line, both in basal conditions (inhibition: 88.3% vs. control) and in the presence of TNF-α (inhibition: 80.9% vs. control) (Figure 6A). In basal conditions, 1 mg/mL and 2 mg/mL FB added alone to the cells, were either equally active or significantly more active than SFN (1 and 2 mg/mL FB: 87.9 and 93.7% inhibition, respectively, vs. control). Moreover, a significantly and even higher inhibition was achieved as 1 mg/mL FB and 10 µM SFN were added together to the culture medium (10 µM SFN vs. 10 µM SFN +1 mg/mL FB, *p* < 0.001). Instead, the addition of 10 µM SFN did not enhance further the maximal inhibitory effect exerted by FB added alone at the highest concentration (2 mg/mL). Interestingly, however, in the presence of TNF-α, SFN combined with FB at the highest concentration (2 mg/mL) was able to completely suppress the production of MMP-3, reduced to an undetectable amount (Figure 6A). The CI was found to be 0.980 and thus, also in this case, the effect of the combined addition of SFN and FB can be considered synergistic. Overall, these results suggest that the SFN and FB combinations could have the potential of preventing the MMP-induced alterations to aged skin, especially if they are inflammation-related.

Figure 6B shows the effect of SFN and FB at the highest concentrations (10 µM and 2 mg/mL, respectively) on the production of IL-1β, a cytokine known to possess a key role in the pro-inflammatory conditions leading to intrinsic skin aging [30]. We observed that both SFN and FB were able to significantly (*p* < 0.05) inhibit IL-1β secretion in normal keratinocytes (inhibition vs. control: 10 µM SFN 71%, 2 mg/mL FB 40%, SFN + FB 89.9%). However, although the SFN/FB combination showed a tendency to markedly inhibit IL-1β production, in our experimental conditions this effect was not significantly higher with respect to the single compounds administered alone (Figure 6B). Similarly, when IL-1β secreted by keratinocytes was analyzed following the exposure to a pro-inflammatory stimulus (TNF-α), both SFN and FB were found to be extremely efficacious in inhibiting cytokine production (inhibition vs. control: 10 µM SFN 87.6%, 2 mg/mL FB 86.1%, SFN + FB 86.8%, *p* < 0.001). Again, in this case the inhibitory effect exerted by the SFN/FB combination did not differ significantly from that of the single compounds alone (Figure 6B). Overall. the results obtained in NCTC 2544 normal keratinocytes in terms of reduced production of MMP-1, MMP-3 and IL-1β, after the treatment with SFN and FB, alone and in combination, are remarkable also in view of the observation that, in these culture conditions, the two compounds did not reduce cell number, but actually increased it. In fact, preliminary experiments demonstrated that both SFN and FB increased keratinocyte number after a 24 h treatment (in particular, by 29.2% vs. control in the presence of 1 mg/mL FB + 10 μM SFN and by 20% in the presence of 2 mg/mL FB + 10 μM SFN). A similar effect was also observed after the addition of 20 ng/mL TNF-α as a pro-inflammatory stimulus (cell growth increase vs. control: 22% and 13%, in the presence of 1 mg/mL FB + 10 μM SFN and 2 mg/mL FB + 10 μM SFN, respectively).

### 3.6. Effect of SFN/FB Combination on Normal Keratinocyte ROS Production

Next, we sought to investigate if the potential antioxidant activity of SFN/FB combination could vary depending on the degree of differentiation of the keratinocytes used. We had previously shown [31] that the molecular features of the two human immortalized keratinocyte cell lines HaCaT and NCTC 2544 made them similar to the differentiated cells present in the superficial layer of the epidermis (HaCaT) or to the undifferentiated cells of the basal layer of the epidermis (NCTC 2544), respectively. Therefore, we investigated (Supplementary Figure S1) the antioxidant activity of FB and SFN, alone and in combination, by analyzing intracellular ROS production of the two keratinocyte lines. Supplementary Figure S1 shows the effects of 2.5–10 µM SFN and 1 mg/mL FB given alone and/or in combination to HaCaT keratinocytes. It should be noted that in all these experiments the maximal concentration of FB (2 mg/mL) was never used, since it inhibited completely ROS production and did not allow the investigation of the possibility that the combination could improve the effect exerted by each single compound. In basal conditions (Supplementary Figure S1A), SFN given alone was able to significantly inhibit ROS production (*p* < 0.05) only at the highest concentrations used (5 and 10 µM SFN, 50.7 and 60.9% inhibition vs. control, respectively). In the presence of a pro-oxidant stimulus (100 µM hydrogen peroxide, H_2_O_2_) (Supplementary Figure S1B), 2.5 µM SFN also significantly inhibited ROS production (42.3% inhibition vs. control, *p* < 0.05). A significant inhibitory effect was observed with 1 mg/mL FB in both the experimental conditions (basal conditions: 69.2% vs. control, *p* < 0.05; + H_2_O_2_: 81.2% vs. control, *p* < 0.001) (Supplementary Figure S1A,B). This effect was higher than that of SFN alone, irrespective of the concentrations of SFN used. Moreover, in basal conditions, only the addition of the highest SFN concentration (10 µM) increased significantly (*p* < 0.05) the effect observed in the presence of 1 mg/mL FB, and the effect resulted to be synergistic (CI = 0.968, see box enclosed in Supplementary Figure S1A). Instead, in cells exposed to the pro-oxidant stimulus (H_2_O_2_), SFN was able to significantly improve (*p* < 0.05) the effect of 1 mg/mL FB, at all the concentration tested (2.5, 5 and 10 µM) (Supplementary Figure S1B). However, when we calculated the CI for the maximal inhibitory effect, obtained with 1 mg/mL FB combined with 10 µM SFN, we found a value of 1 (see box enclosed in Supplementary Figure S1B), indicating that under these pro-oxidant conditions the effect of the SFN/FB combination was only additive.

In basal conditions, similarly to what was observed in HaCaT cells, cell ROS production was significantly inhibited (Supplementary Figure S1C) in NCTC 2544 by either SFN added alone at the highest concentrations (5 and 10 µM SFN: 65.7% and 64.9% inhibition vs. control, *p* < 0.05, respectively) or FB added alone at 1 mg/mL (59.8% inhibition vs. control *p* < 0.01). However, in NCTC 2544 keratinocytes, different from what was found in HaCaT cells in the same basal conditions, the inhibitory effect exerted by FB alone was never increased by the addition of SFN, and thus, it was not possible to evaluate the CI of the two compounds.

In NCTC 2544 cells cultured in the presence of the pro-oxidant stimulus, differently from what was observed for the same cells in basal conditions and for the HaCaT cells in the identical pro-oxidant conditions (see Supplementary Figure S1C,B, respectively), SFN, individually added, significantly inhibited ROS production only if added at the highest concentration (10 µM SFN: 50.5% inhibition vs. control, *p* < 0.05) (Supplementary Figure S1C). On the contrary, similarly to what was observed in the other cells and conditions, FB added alone at 1 mg/mL was able to significantly inhibit ROS production in NCTC 2544 cells cultured in the presence of H_2_O_2_ (65.7% inhibition vs. control *p* < 0.001). However, differently from what was observed with the more differentiated HaCaT cells, a significantly increased inhibitory effect (*p* < 0.05) on ROS production was observed in NCTC 2544 cells cultured in the pro-oxidant conditions only if the highest concentrations of SFN (5 µM or 10 µM, but not 2.5 µM SFN) were combined with 1 mg/mL FB. (Supplementary Figure S1D). The calculated CI for FB combined with 10 µM SFN in this condition was 0.971 (box enclosed in Supplementary Figure S1D), indicative of a synergistic effect. It should be noted that, in all these experiments, the maximal concentration of FB (2 mg/mL) was never used, since it completely inhibited ROS production and did not allow the investigation of the possibility that the combination could improve the effect exerted by each single compound. Overall, these findings indicate that the more differentiated HaCaT cells were always more sensitive than the NCTC 2544 cells to the activity of SFN alone and to the SFN/FB combination.

## 4. Discussion

In the present work we have demonstrated the advantages that may result from combining SFN, a sulfur-isothiocyanate present in vegetables such as broccoli or cauliflowers, with FB, the patented extract of the tropical fern *Polypodium leucotomos,* in a unique dietary supplement. We were interested in the SFN/FB combination since it is present in a dietary supplement already on the market. Our results have demonstrated that the SFN/FB combination is able to significantly and synergistically inhibit melanoma cell growth in vitro, as well as its invasive and angiogenic potential. Moreover, we have observed that the combined administration of SFN and FB to normal skin cells in vitro increased significantly and in a synergistic manner the inflammation-induced MMP production and the antioxidant activity of the individual compounds.

Several reports have demonstrated that skin cells and tissues benefit from the use of SFN, a sulfur-isothiocyanate present in vegetables such as broccoli or cauliflowers [20]. Similar evidence was provided for FB, the patented extract of the tropical fern *Polypodium leucotomos* [10,11], since both the substances exert powerful antioxidant, anti-inflammatory and antineoplastic activities [10,15,16,17,18,19,20]. In the present study, we demonstrated that a combination of these two compounds was able to inhibit the growth of the two human melanoma cell lines WM115 and WM266-4 significantly more than the two compounds administered alone, and in a synergistic manner. This is worth noticing, since a dietary supplement combining the two substances have already been developed, and it is currently recommended for the prevention as well as for the adjuvant therapy of several skin disorders. However, so far, a direct experimental demonstration of the actual advantage that may derive for skin cells from the use of such a combination has not been provided. Moreover, the synergistic growth inhibitory effect of the SFN/FB combination suggests a potential additional use for the already existing dietary supplement containing this formulation as a preventive measure for melanoma. This tumor is an extremely malignant cancer showing a high invasive and metastatic potential [32] and active migration of cancer cells is an essential step to invade and metastasize [33]. Our finding that SFN may inhibit migration of different melanoma cell lines, even though at different degrees, is not in agreement with the observation of Pradhan et al. [34], who did not find any effect of SFN on B16F10 melanoma cell migration. However, this discrepancy may be related to the different experimental conditions used, since these authors used murine melanoma cells, exposed them to an SFN concentration (20 µM) much higher than that used by us, and the exposure was shorter (16 h). However, more recently Arcidiacono et al. [19] found a similar effect of SFN on cell migration in different human melanoma cells (A375) by exposing the cells for 24 h to 2 μg/mL SFN (which corresponds to approximately 11 μM SFN), a concentration very similar to that we used. In the present study, we have demonstrated for the first time that FB has the potential to powerfully reduce melanoma cell migration. In fact, when it was added at the maximal concentration (2 mg/mL), it was able to almost completely inhibit melanoma cells wound healing in vitro for all the duration of the experiment (72 h). Even more interesting, we demonstrated that, when SFN was combined to FB added at the lowest concentration (1 mg/mL), the inhibitory effect on migration was increased to near maximal levels and the effect was synergistic. This finding suggests that a combination of SFN and FB could allow the reduction of the doses of the individual bioactive compounds to obtain healthy effects, and further corroborates the hypothesis that this combination has the potential to be used as a powerful protective agent against melanoma development and progression.

The inhibiting action of FB alone on MMP production observed by us in this study confirmed the finding of Philips et al. [35], who exposed dermal fibroblasts and melanoma cells to an extract of *Polypodium leucotomos*. Zamarron et al. also found that Fernblock, at concentrations very similar to those used in our study (0.5 and 1 mg/mL), was able to inhibit the production of MMP-1 in dermal fibroblasts exposed to infrared A radiation [36]. The SFN inhibiting action on MMP-9 is also consistent with the finding of Pradhan et al. [34]. Moreover, our finding is also in line with the observation that the production of MMP-9 by melanoma cells may be regulated by antioxidants added externally [37]. Our data are also in agreement with reports showing that the production of MMP-1 and MMP-2 (and the modulation of the invasive potential of melanoma cells) may be dependent on changes in the activities of enzymatic systems involved in the production of ROS and RNS (i.e., in the modulation of cellular oxidative stress) [38,39]. Finally, our findings on the inhibitory effects exerted on SFN and FB alone and in combination on MMP production suggest that the inhibitory effect on melanoma cell migration exerted by both the compounds was strictly related to the inhibition of the MMPs. Moreover, the observation that SFN and FB exert a synergistic inhibitory effect on the secretion of MMP-1, -2 and-3 further corroborates the potential use of the SFN/FB combination for the prevention of invasion and metastasis during melanoma progression.

We have also demonstrated here for the first time that FB is able to inhibit inflammasome activation. Moreover, our results confirm that SFN can inhibit NLRP3 inflammasome activation as previously observed by using other experimental models in different pathological conditions [40,41,42,43]. Our finding is also in line with the recent observation [44] that ferulic acid, a main constituent of FB (*Polypodium leucotomos* extract), when administered to a rat model of auto-inflammation, was able to inhibit the formation of inflammasome. It appears also of great interest that in WM266-4 melanoma cells the SFN/FB combination inhibited inflammasome formation (i.e., NLRP3, cleaved caspase-1 and ASC expression) and IL-1β production significantly more than each single compound. In particular, the observed synergistic effect of this combination suggests its potential use for the prevention of the inflammatory microenvironment, a key step in melanoma development. Moreover, the inhibiting effect of both SFN and FB on the production of the pro-angiogenic factor VEGF demonstrated in metastatic melanoma cells is in agreement with what was previously observed by using SFN alone in other cancer cell models in vitro*,* including colon [45], prostate [46,47], tongue squamous [47] cancer cells, as well as leukemia [48] and hepatocellular carcinoma cells [49]. However, in all these works, SFN was able to efficiently inhibit neo-angiogenesis only at concentrations (12.5–50 µM) much higher than those found to be effective in our experimental model (5–10 µM). Furthermore, to the best of our knowledge, this is the first report demonstrating the potential anti-angiogenetic effect of FB in metastatic melanoma cells, as well as the significantly enhanced and synergistic anti-angiogenetic effect of the SFN/FB combination with respect to the single compounds. This finding also corroborates the hypothesis that the SFN/FB combination could represent a new interesting possibility for melanoma prevention.

Of interest also is the finding that the SFN/FB combination can reduce the production of the pro-inflammatory cytokine IL-1β and those of MMP-1 and MMP-3 in the normal human keratinocyte cell lines. In fact, an inflammatory response leading to MMP activation, and the consequent extracellular matrix structural and functional alterations, have been involved in the development and progression of skin aging [50]. Moreover, inflammation has also been involved in the development and progression of keratinocyte-derived tumors of the skin [51,52]. Our observation that SFN is protective against photoaging through a mechanism involving the production of MMP-1 confirm the results of Chaiprasongsuk et al. [53] obtained in mouse skin exposed to UVA radiation. Moreover, the observed synergistic effect of the SFN/FB combination on MMP-1 production in normal keratinocytes is interesting since it suggests the potential use of this combination for inclusion in a dietary supplement for sustained prevention of photoaging.

Even though we have not found any synergistic effect of the SFN/FB combination on the production of IL-1β in normal keratinocytes, we observed an inhibitory effect of FB administered individually to both normal and neoplastic skin cells, thus confirming a finding previously obtained [54] in mononuclear cells isolated from healthy donors and exposed in vitro to both LPS or PHA and to a *Polypodium leucotomos* extract. The inhibitory effect of SFN given alone on IL-1β production is also in line with what was reported previously in different experimental models, including murine melanoma cells [18].

Furthermore, we investigated if the effect of SFN and FB on MMP-1 observed by us could be related to an inhibition of ROS production in HaCaT and NCTC 2544 keratinocytes exerted by the two compounds. In fact, previously, Kim et al. [55] observed that ROS-induced MAPK/AP-1 signaling was associated with an increased secretion of MMP-1 in HaCaT keratinocytes and in dermal fibroblasts. Moreover, it has been reported that SFN acts as an indirect antioxidant, since it powerfully induces endogenous cellular defenses regulated by transcription factor nuclear factor erythroid 2-related factor 2 (Nrf2), which in turn acts to regulate the expression of cellular antioxidant enzymes [56]. We found that in both the keratinocytes, SFN and FB administered individually were able to exert a powerful inhibiting effect on ROS production, either in basal conditions or in the presence of a pro-oxidant stimulus (H_2_O_2_). These findings agree with previous results showing that FB may exert its photoprotective effects by an antioxidant mechanism (for a review, see [11]). Moreover, we observed that the inhibiting effect on ROS production was markedly enhanced when both the keratinocytes were treated with a combination of SFN and FB. However, the SFN/FB combination inhibited ROS production always significantly, in a synergistic manner and at lower SFN concentrations only in the more differentiated HaCaT keratinocytes, representing a cell model of the superficial layer of the epidermis. This observation is of great interest, given that skin surface is particularly exposed to the oxidative action of ultraviolet radiation. Thus, these findings suggest that a dietary supplement containing SFN and FB could provide a more consistent protection than the occasional topic application of antioxidants contained in sunscreens and other skin preparations.

## 5. Conclusions

The results obtained in this study suggest that SFN and FB delivered together in a dietary supplement could have the potential to exert powerful and synergistic protective effects against oxidative, pro-inflammatory and carcinogenic insults to skin, and to impede the development of skin aging, as well as the progression of melanoma. A further interesting development of this work to gain new mechanistic insight into this subject could be the evaluation of the ability of SFN, FB and their combinations to modulate the epigenetic regulation of the expression of the genes codifying for pre-IL-1β, VEGF or MMPs in skin cells. In fact, the ability of phytochemical and nutritional factors to modify the epigenoma represents currently one of the newest and most exciting areas of nutrition research [57,58,59]. In conclusion, we would like to point out that the findings reported here are applicable to a dietary supplement combining SFN and FB, which is already present on the market and recommended by dermatologists for the prevention and the adjuvant therapy of several skin disorders. This, in our opinion, is important since, so far, direct experimental demonstrations of the actual advantages that may derive using specific combinations of bioactive compounds in the formulation of dietary supplements are not required by law and, thus, seldom provided. On the contrary, experimental works of this kind and their outcomes would allow an evidence-based approach for treatments with dietary supplements (sometimes prescribed also for long periods of time). They would permit us also to overcome the current prejudiced belief that the combination of two or more bioactive compounds in a unique dietary supplement may only increase their individual efficacy and never induce deleterious effects.

## Supplementary Materials

The following are available online at https://www.mdpi.com/2072-6643/12/6/1608/s1, Figure S1: Effect of a 24 h-treatment with different concentrations of SFN (2.5–10 µM) and FB (1 mg/mL), alone and in combination, on ROS production in HaCaT and NCTC 2544 keratinocytes in basal conditions and in the presence of 100 µM H_2_O_2_ as a pro-oxidant stimulus. (*: significantly different from respective control, *p* < 0.05, One-way ANOVA followed by Tukey’s test). In panels A, B and D, is shown the CI obtained following the treatment with SFN and FB (10 μM SFN and 1 mg/mL FB). The interaction was considered significant when *p* < 0.05 (2 × 2 factorial ANOVA) (see Materials and methods section for details).

## Abbreviations

ASC	Adaptor Protein Apoptosis-Associated Speck-Like Protein Containing CARD
DCF-DA	6-carboxy-2′,7′-dihydro-dichlorofluorescein diacetate
DMEM	Dulbecco’s Modified Eagle’s Medium
DMSO	dimethylsulfoxide
SFN	sulforaphane
FB	Fernblock^®^
IL-1β	interleukin-1β
MMP	metalloproteinases
NLRP3	NACHT, LRR and PYD domains-containing protein 3
PBS	phosphate buffered saline
ROS	reactive oxygen species
TNF-α	tumor necrosis factor-α
VEGF	vascular endothelial growth factor
