# Supplementary figures and images for: The Combination of Sulforaphane and Fernblock® XP Improves Individual Beneficial Effects in Normal and Neoplastic Human Skin Cell Lines
Source: Nutrients. 2020 May 30;12(6):1608. doi: 10.3390/nu12061608 (PMC7353001; doi:10.3390/nu12061608)

## HaCaT

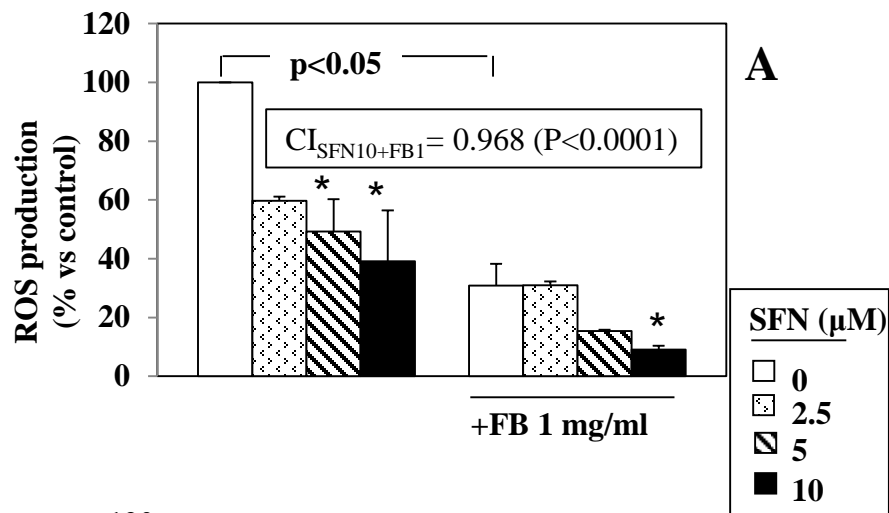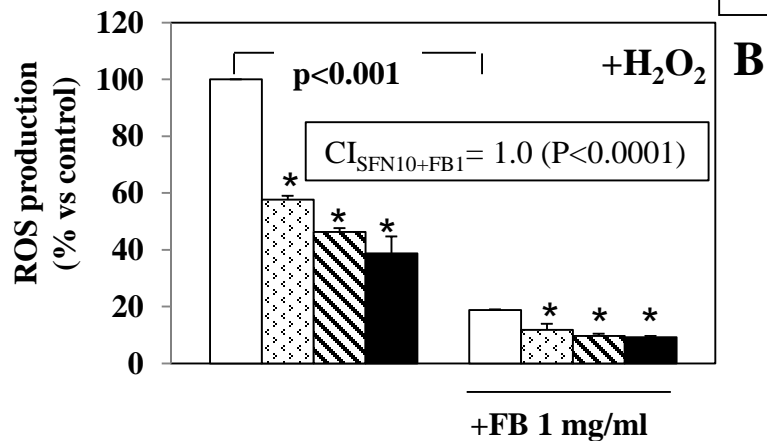

## NCTC 2544

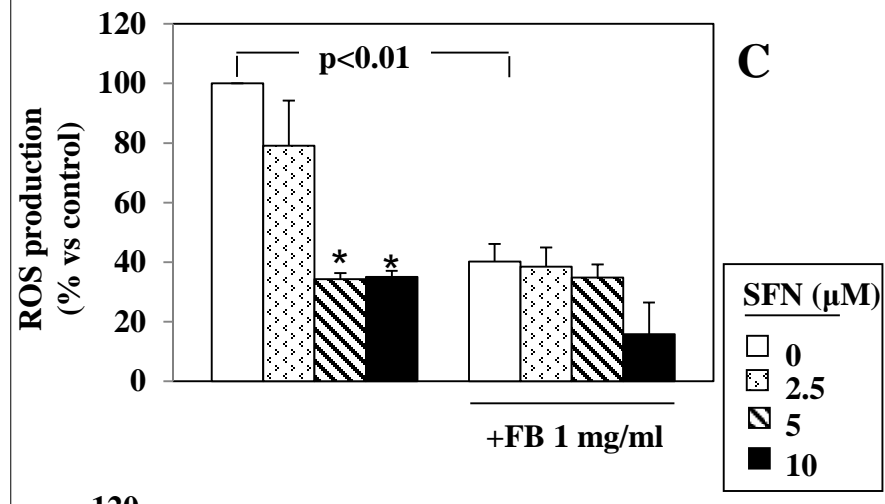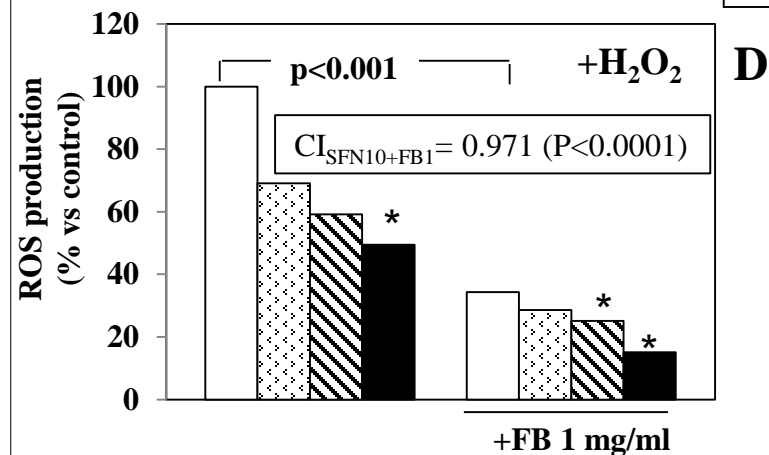

Supplement: Supplementary file 1 [file nutrients-12-01608-s001.pdf]
